# Case Report: Intravascular papillary endothelial hyperplasia in the superficial temporal artery

**DOI:** 10.3389/fsurg.2025.1556819

**Published:** 2025-03-26

**Authors:** Rui Cong, Shasha Shi, Yang Li, Jianyuan Xuan

**Affiliations:** Department of Ultrasound, The Second Hospital of Dalian Medical University, Dalian, China

**Keywords:** intravascular papillary endothelial hyperplasia, vascular tumor, superficial temporal artery, ultrasonographic features, diagnosis

## Abstract

Intravascular papillary endothelial hyperplasia is a rare benign vascular lesion that typically occurs in thrombosed dilated veins or vascular tumors, with its occurrence within arterial blood vessels being uncommon. In this report, we present a case of intravascular papillary endothelial hyperplasia within superficial temporal artery in a 35-year-old Chinese male patient. The patient initially noticed a non-tender pulsatile mass in his left temple three years ago, which has gradually increased in size over time. Ultrasound examination revealed a well-defined, hypoechoic oval lesion in the left temporal region, with a blood vessel traversing through it and connecting to the superficial temporal artery. Complete excision of the mass was performed, and histological examination confirmed intravascular papillary endothelial hyperplasia. We provide an extensive literature review on intravascular papillary endothelial hyperplasia tumor along with a discussion on ultrasound findings.

## 1 Introduction

Intravascular papillary endothelial hyperplasia (IPEH), also known as Masson's tumor, is a rare benign vascular endothelial abnormal proliferative lesion, accounting for 2%–4% of vascular tumors of the soft tissue (1). It Initially described by Pierre Masson in 1923 as an intravascular vegetating hemangio-endothelioma, the current name of IPEH was proposed by Clearkin and Enzinger in 1976 (2).

IPEH can mimic various benign tumors (cysts, haemangiomas and lipomas) and malignant tumors (angiosarcomas, melanoma and squamous cell carcinomas) and it lacks a distinct or distinguishing clinical and radiological appearance (3). Definitive diagnosis relies on histopathological examination, which is characterized by reactive proliferation of papillary endothelial cells and surgical excision is the preferred and most effective treatment for IPEH (4).

In this case report, we present a patient diagnosed with IPEH in the superficial temporal artery (STA). At presentation, the patient exhibited a pulsatile swelling approximately 2 cm in size in the left temporal region. Ultrasound imaging revealed the STA traversing through the tumor, with both color Doppler and spectral Doppler furnishing comprehensive details. Given the scarcity of similar cases reported in literature, particularly those with comprehensive ultrasound documentation, this case is presented to enhance awareness of the distinctive ultrasound features for diagnosing IPEH.

## 2 Case description

**Figure 1:**
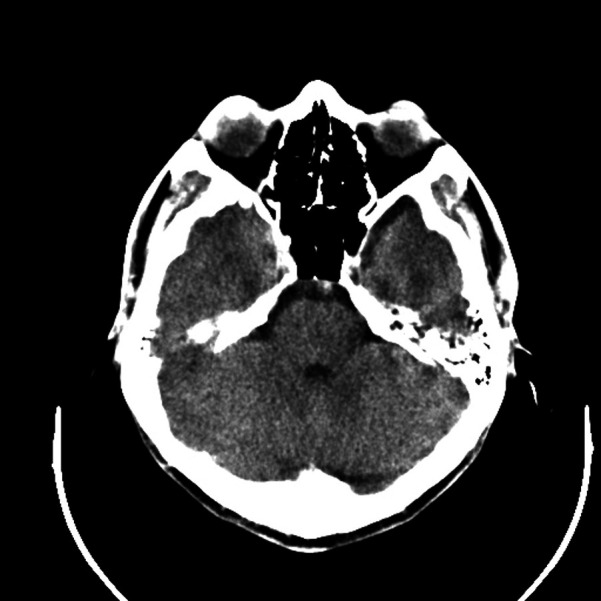
Head CT scan revealed a benign lesion located in the left temporal region.

**Figure 2:**
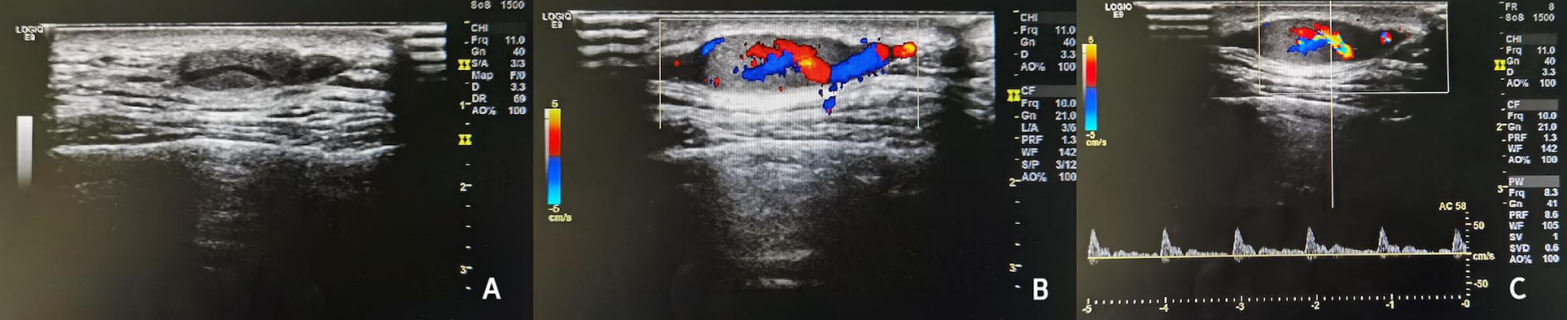
Ultrasound images. **(A)** The B-mode image showed a well-defined, hypoechoic oval lesion within which a tubular anechoic structure was observed extending from bottom to top. **(B)** The Color Doppler imaging exhibited pulsatile color flow signals originating from the superficial temporal artery. **(C)** The Spectral Doppler demonstrated continuous arterial blood flow.

**Figure 3:**
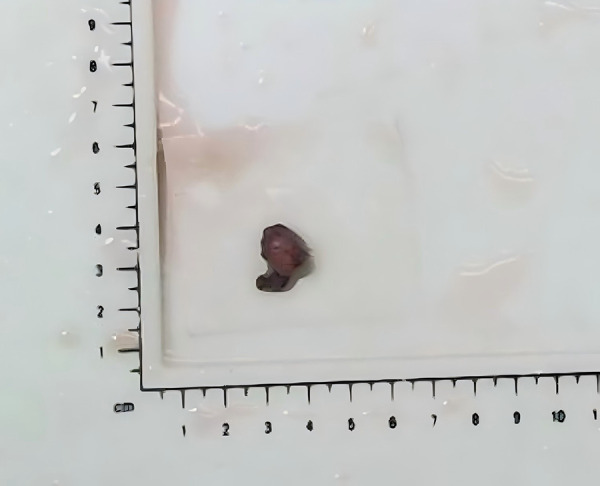
Postsurgical image of the lesion. Postoperative pathology revealed a small nodule measuring 1.2 × 0.8 × 0.6 cm in diameter, with a grayish-red appearance and a soft texture.

**Figure 4:**
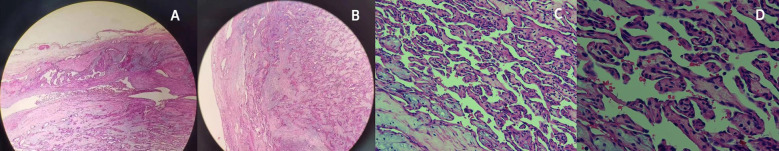
Artery section demonstrating papillae covered by endothelial cells with homogeneous nuclear chromatin and no evident atypia; fibrovascular stroma interspersed among the papillae. Hematoxylin and eosin staining **(A** and **B)** × 100, **C** × 200 and **D** × 400.

A 35-year-old man visited the Department of Aesthetic Plastic Surgery for a pulsating swelling on his left forehead. He first noticed it three years ago and underwent a head CT scan at that time, which revealed a benign lesion in the left temporal region, as shown in Figure 1. He observed that the swelling had been gradually increasing in size, so he intended to undergo surgical excision. He reported no other clinical symptoms, such as pain, and had no definitive history of head or face trauma. Physical examination revealed a pulsatile swelling of approximately 2 cm in the left temporal region of the patient. The mass was mobile, firm, and not adherent to surrounding tissues. It was non-tender and had a palpable pulse. The skin temperature and color around the mass were normal. The ultrasound examination revealed a well-defined, hypoechoic oval lesion measuring 1.8 cm × 0.6 cm in the subcutaneous region of the left temporal lobe. Within this lesion, a tubular anechoic structure was observed extending from bottom to top of the lesion and directly connecting to the STA. The echogenicity surrounding the tubular anechoic structure was homogeneous. Color Doppler and spectral Doppler imaging revealed color flow signals and arterial flow spectra within the tubular anechoic structure, which originated from the STA. No blood flow signals were detected outside around the tubular anechoic structure. Detailed ultrasound images are shown in Figures 2A–C. It was dissected free of the surrounding tissue and excised under local anesthesia. The postoperative pathology revealed a small nodule measuring 1.2 × 0.8 × 0.6 cm in diameter, exhibiting a grayish-red appearance on the cut surface and possessing a soft texture (Figure 3). The histopathological findings confirmed that it was consistent with the benign nature of IPEH and no features suggestive of malignancy were observed (Figures 4A–D). The timeline of the related operations and findings is presented in Table 1. Following surgical excision, the surgical site exhibited excellent healing without any complications such as bleeding, infection, or nerve damage. The surgical site healed well, and there were no adverse effects on the patient's appearance or function. Moreover, no recurrence was observed during the 24-month follow-up period post-surgery.

**Table 1:** Timeline.

Time stage	Related operations and findings
Three years ago	The patient first noticed a pulsating swelling on his left forehead. At that time, a head CT scan was performed, which revealed a benign lesion in the left temporal region (as shown in Figure 1).
Recently	The patient observed that the swelling had been gradually increasing in size and decided to undergo surgical excision. Physical examination showed a pulsatile swelling of approximately 2 cm in the left temporal region. The mass was mobile, firm, not adherent to surrounding tissues, non-tender and had a palpable pulse. The skin temperature and color around the mass were normal.
	Ultrasound examination revealed a well-defined, hypoechoic oval lesion measuring 1.8 cm × 0.6 cm in the subcutaneous region of the left temporal lobe. Within this lesion, a tubular anechoic structure was observed extending from bottom to top. Color Doppler and Spectral Doppler imaging exhibited color flow signals and arterial flow spectra originating from the superficial temporal artery (detailed ultrasound images can be seen in Figures 2A–C).
During the operation	The lesion was dissected free of the surrounding tissue and excised under local anesthesia.
After the operation	Postoperative pathology revealed a small nodule measuring 1.2 × 0.8 × 0.6 cm in diameter, with a grayish-red appearance on the cut surface and a soft texture (Figure 3). Histopathological findings confirmed that it was consistent with intravascular papillary endothelial hyperplasia (Figures 4A–D).

## 3 Discussion

This study presents a case of IPEH occurring in the STA, which is a rare occurrence. Christopher et al. previously reported a patient who presented with IPEH associated with an arteriovenous malformation of the radial artery (5). Another case reported the primary form of Masson's tumor in an aneurysmal ulnar artery (6). Limited literature exists on IPEH within the STA, with only two cases reported in an aneurysm of the STA (7, 8), both lacking ultrasound images. While most publications describe sonographic appearances of IPEH within veins, reports on its appearance within arteries are scarce. Additionally, due to their small size and superficial location, most cases of IPEH are not imaged, preoperative imaging has been utilized only in a few cases (9–11).

The echogenicity on ultrasound was classified as hypoechoic, isoechoic, or hyperechoic based on comparison with skeletal muscle. Additionally, color Doppler images revealed distinct vascularity patterns categorized as central, septal, or peripheral. Prior reports have suggested that IPEH typically appears as well-defined, homogeneous, hypoechoic and hypervascular (9, 10). Oleg et al. (9) proposed that the grayscale appearance of the IPEH was indicative of an adherent thrombus, while color Doppler sonography demonstrated its hypervascular nature. Similarly, Lee et al. (10) suggested that the hypoechoic portion observed on ultrasound correlated histopathologically with thrombi, and presence of hyperechoic septa and central portion containing vascularity on US corresponded to hypertrophic papillary epithelium and a fibrovascular core. Kim et al. (11) further indicated that vascularity of subcutaneous IPEH may correlate not only lesion size but also size and/or number of intralesional vascular channels.

Notably, Kim et al. (11) emphasized that the internal septum-like structures and the origin vessel connecting to the lesion represent relatively characteristic US features of subcutaneous IPEH. These septa likely correspond histologically to fibrin-rich connective tissue in in organizing thrombi. This heterogeneity distinguishes IPEH from other vascular masses, such as angioleiomyoma or non-subungual glomus tumors, which typically display homogeneous echotexture. However, both IPEH and cavernous hemangiomae exhibit heterogeneous echogenicity along with septa or mesh-like strucures, rendering this overlapping feature unreliable for differentiation.

The origin vessel connecting to the lesion is an important differentiating point. In Kim's study (11), origin vessels connecting to the lesion were observed in 40% (4/10) cases, yet none demonstrated continuity with the origin vessel. A representative case —a 21-year-old female with pure IPEH in her had—showed a solitary intralesional vascular channel devoid of origin vessel connection. The arterial or venous nature of this channel was not clearly identified. The absence of detection of the penetrating vessel may be attributed to low blood flow dynamics typically associated with venous vessels.

The typical ultrasound feature of vascular penetration observed in this study exhibits the characteristics mentioned above, which is a more characteristic ultrasound manifestation. In the two previously reported cases of IPEH within the STA, only one case underwent ultrasound examination which revealed turbulent flow within the aneurysm along with a mural thrombus (7). However, these ultrasound features are not specific to the diagnosis of IPEH and it is impossible to determine the original vessel. In contrast, the presence of penetrating vessel can identify the original vessel and may serve as a valuable specific manifestation for diagnosing IPEH. Nevertheless, further validation through larger case series is necessary to confirm this observation.

IPEH is histologically classified into three main types according to Hashimoto et al. (12). The primary type is typically associated with a dilated vein but may less commonly be associated with an artery. The secondary type resides within a preexisting vascular malformation (usually a cavernous hemangioma, pyogenic granuloma or lymphangioma). The extravascular type, which is the least common, arises in organizing extravascular hematomas. Previous retrospective studies have suggested that the primary type is the most common subtype in the population (3).

Chapman et al. (8) proposed that when these tumors involve the arterial system rather than the more common venous presentation, they are almost universally associated with aneurysmal dilation. In our case, the STA affected exhibited a width of approximately 0.6 cm, indicating significant dilation compared to the normal diameter of STA. This suggests that in this case, there should also be associated aneurysmal dilation in the STA as suggested by Chapman et al.'s study. However, Chapman et al. (8) classified their patient as secondary type (arising in STA aneurysm), differs from our understanding in this study where we classify our case as primary type (arising in dilated STA). This discrepancy may arise from divergent interpretations regarding the inclusion of preexisting vascular malformation within the classification. Our study does not consider aneurysmal dilation as part of vascular malformation, instead, it represents a form of vascular dilation similar to dilated veins. Our case should still be classified as primary type.

The pathogenesis of IPEH remains controversial. It is hypothesized that IPEH might be associated with an atypical process of thrombus organization. Similar to the process of thrombus organization and recanalization, the developmental pathogenesis of IPEH lesion formation occurs in several steps. In the early stage, endothelial cells become embedded within the thrombus, characterizing the early lesions. Subsequently, the multiplying endothelial cells partition the thrombus digested by collagenase into irregular aggregates, out of which papillary structures develop. In the final stage, these papillae coalesce and give rise to anastomosing vascular structures (13). This hypothesized mechanism is consistent with the analysis of the correlation between ultrasound and pathological findings mentioned earlier. This mechanism has the potential to account for the distinctive ultrasound features noted in the present case, which share similarities with recanalization following venous thrombosis but also exhibit notable differences. Unlike post-thrombotic recanalization, the vascular channels herein are more regular. Moreover, this mechanism can also account for the differences present in the sonogram. The distinctive ultrasound features noted in this particular case have scarcely been documented, perhaps due to varying stages of lesion development, as in some instances, the stage of anastomosing vascular structures formation has not yet been reached.

The ultrasound images in this study exhibit typical characteristics, distinctly visualizing vascular penetration. These images are rare and have not been seen as complete and typical ultrasound images in know reports. IPEH lacks distinct clinical and radiological features, and histopathological examination remains the only definite method. According to previous literature, surgical excision is the most effective treatment for IPEH. However, this distinctive ultrasonic feature may significantly aid in the preoperative diagnosis of IPEH and potentially facilitate the exploration of alternative treatments, such as radiotherapy.

## Data Availability

The raw data supporting the conclusions of this article will be made available by the authors, without undue reservation.

## References

[1] Kreutner A Jr Smith RM Trefny FA. Intravascular papillary endothelial hyperplasia. Light and electron microscopic observations of a case. Cancer. (1978) 42:2304–10. 10.1002/1097-0142(197811)42:5<2304::AID-CNCR2820420530>3.0.CO;2-G 569010

[2] Clearkin K Enzinger F. Intravascular papillary endothelial hyperplasia. Arch Pathol Lab Med. (1976) 100:441–4. 947306

[3] Yang K Pan CX Russell-Goldman EE Nambudiri VE. Characterization of intravascular papillary endothelial hyperplasia: a multicentre cohort. Clin Exp Dermatol. (2022) 47(8):1550–3. 10.1111/ced.15182 35297528

[4] Abdo EM Farouk N Elshinawy WE Mohamed Ahmed E Raafat MA Husien Abdo W Masson’s tumor as an uncommon cause of neck mass: a case presentation. Vasc Endovascular Surg. (2024) 58:405–9. 10.1177/15385744231215102 37962479 PMC10996301

[5] Stark C Olsen D Morris C Bertges D Najarian K. Intravascular papillary endothelial hyperplasia (Masson’s tumor) of the radial artery: a case report. Cardiovasc Intervent Radiol. (2016) 39:1658–61. 10.1007/s00270-016-1410-6 27402306

[6] Chang K Barlaben A Farley S. Masson’s tumor in the ulnar artery. J Vasc Surg. (2012) 56:223–5. 10.1016/j.jvs.2012.01.010 22387264

[7] Shuji M Ryuji K Hisashi S Ken O Toshiharu S Mutsuro T Intravascular papillary endothelial hyperplasia in an aneurysm of the superficial temporal artery: report of a case. Surg Today. (2011) 41:1450–4. 10.1007/s00595-010-4499-2 21922377

[8] Chapman SC Zak PW Scaife M Murdoch G Eslami MH. Masson tumor (intravascular papillary endothelial hyperplasia) arising in a superficial temporal artery aneurysm. J Vasc Surg Cases Innov Tech. (2019) 5:388–91. 10.1016/j.jvscit.2019.02.013 31517158 PMC6727241

[9] Lysyy O Schwartz I Kolander Y Strauss S. Sonographic features of intravascular papillary endothelial hyperplasia (Masson’s tumor) in the forearm. J Clin Ultrasound. (2011) 39:301–3. 10.1002/jcu.20760 21547933

[10] Lee SJ Choo HJ Park JS Park Y-M Eun CK Hong SH Imaging findings of intravascular papillary endothelial hyperplasia presenting in extremities: correlation with pathological findings. Skeletal Radiol. (2010) 39:783–9. 10.1007/s00256-010-0888-2 20157705

[11] Kim OH Kim YM Choo HJ Lee SJ Kim YM Yi JH Subcutaneous intravascular papillary endothelial hyperplasia: ultrasound features and pathological correlation. Skeletal Radiol. (2016) 45:227–33. 10.1007/s00256-015-2281-7 26559670

[12] Hashimto H Daimaru Y Enjoji M. Intravascular papillary endothelial hyperplasia A clinicopathologic study of 91 cases. Am J Dermatopathol. (1983) 5:539–46. 10.1097/00000372-198312000-00004 6666836

[13] Akdur NC Donmez M Gozel S Ustun H Hucumenoglu S. Intravascular papillary endothelial hyperplasia: histomorphological and immunohistochemical features. Diagn Pathol. (2013) 8:1–6. 10.1186/1746-1596-8-167 24125024 PMC4016006

